# Gradient boosting decision-tree-based algorithm with neuroimaging for personalized treatment in depression

**DOI:** 10.1016/j.neuri.2022.100110

**Published:** 2022-11-11

**Authors:** Farzana Z. Ali, Kenneth Wengler, Xiang He, Minh Hoai Nguyen, Ramin V. Parsey, Christine DeLorenzo

**Affiliations:** a Department of Biomedical Engineering, Stony Brook University, Stony Brook, NY, USA; b Department of Psychiatry, Columbia University and New York State Psychiatric Institute, New York, NY, USA; c Department of Radiology, Stony Brook Medicine, Stony Brook, NY, USA; d Department of Radiology, Northshore University Hospital, Manhasset, NY, USA; e Department of Computer Science, Stony Brook University, Stony Brook, NY, USA; f Department of Psychiatry, Renaissance School of Medicine at Stony Brook University, Stony Brook, NY, USA

**Keywords:** Artificial intelligence, Imaging informatics, Medical imaging, FDG PET, Magnetic resonance spectroscopy, XGBoost

## Abstract

**Introduction:**

Pretreatment positron emission tomography (PET) with 2-deoxy-2-[^18^F]fluoro-D-glucose (FDG) and magnetic resonance spectroscopy (MRS) may identify biomarkers for predicting remission (absence of depression). Yet, no such image-based biomarkers have achieved clinical validity. The purpose of this study was to identify biomarkers of remission using machine learning (ML) with pretreatment FDG-PET/MRS neuroimaging, to reduce patient suffering and economic burden from ineffective trials.

**Methods:**

This study used simultaneous PET/MRS neuroimaging from a double-blind, placebo-controlled, randomized antidepressant trial on 60 participants with major depressive disorder (MDD) before initiating treatment. After eight weeks of treatment, those with ≤ 7 on 17-item Hamilton Depression Rating Scale were designated *a priori* as remitters (free of depression, 37%). Metabolic rate of glucose uptake (metabolism) from 22 brain regions were acquired from PET. Concentrations (mM) of glutamine and glutamate and gamma-aminobutyric acid (GABA) in anterior cingulate cortex were quantified from MRS. The data were randomly split into 67% train and cross-validation (*n* = 40), and 33% test (*n* = 20) sets. The imaging features, along with age, sex, handedness, and treatment assignment (selective serotonin reuptake inhibitor or SSRI vs. placebo) were entered into the eXtreme Gradient Boosting (XGBoost) classifier for training.

**Results:**

In test data, the model showed 62% sensitivity, 92% specificity, and 77% weighted accuracy. Pretreatment metabolism of left hippocampus from PET was the most predictive of remission.

**Conclusions:**

The pretreatment neuroimaging takes around 60 minutes but has potential to prevent weeks of failed treatment trials. This study effectively addresses common issues for neuroimaging analysis, such as small sample size, high dimensionality, and class imbalance.

## Graphical Abstract

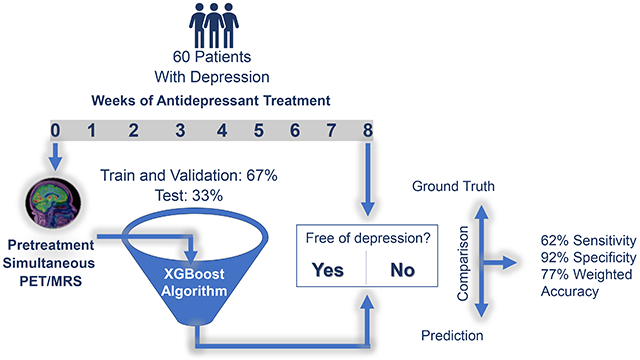

## 1. Introduction

There is an urgent need for reliable prediction of potential antidepressant failure in treatment of major depressive disorder (MDD). Pretreatment electroencephalogram (EEG) has predicted antidepressant efficacy with performance considerable for clinical utility [1]. EEG electrodes are placed on the surface of the brain to obtain functional measures, leading to lower spatial resolution compared to molecular neuroimaging, where the source of signal may not be apparent [2]. Molecular imaging modalities, such as positron emission tomography (PET) with 2-deoxy-2-[^18^F]fluoro-D-glucose (FDG) and proton magnetic resonance spectroscopy (^1^H-MRS or MRS) have been useful for early assessment and risk stratification in patients with neurological, oncological and cardiovascular disorders [3]. Yet, they have not been proved clinically useful due to lack of accuracy [4].

FDG-PET is a sensitive indicator of cerebral function, leading to its extensive use for assessing response to the most widely used first-line MDD treatment, selective serotonin reuptake inhibitor (SSRI) [5]. However, these prior studies do not agree on the predictive region/measure and their prediction could not be replicated using conventional statistics [6].

Glutamine (Gln) is the precursor to the excitatory neurotransmitter, glutamate (Glu) and inhibitory neurotransmitter, *γ*-aminobutyric acid (GABA), and all three are essential amino acids for brain metabolism [7]. Prior magnetic resonance spectroscopy (MRS) studies showed that higher pretreatment Glu in the anterior cingulate cortex (ACC) [8] can predict better response, but other MRS studies showed no predictive potential for antidepressant response using Glu [9,10], GABA [9–11], or Glx (Gln/Glu) [12], adding to the lack of consensus on biomarkers of MDD treatment. These prior inconsistent findings using single imaging modalities with fewer functional features and conventional statistical models have motivated the use of machine learning (ML) with multimodal neuroimaging (both PET and MRS measures) for better prediction accuracy [13].

The purpose of this study was to identify biomarkers to predict remission (absence of depression) after eight weeks of treatment using pretreatment neuroimaging measures from i) PET and ii) MRS with a widely popular (due to superior performance) supervised, gradient tree boosting ensemble algorithm, eXtreme Gradient Boosting (XGBoost), which is a fast, scalable, and explainable artificial intelligence (AI) classifier with strong regularization [14]. XGBoost allows the adjustment of multiple hyperparameters to avoid overfitting and automatically ranks the most predictive features that can be used as biomarkers for remission. This article will have the following contribution regarding predictive biomarkers for antidepressant treatment.

- This study provides a novel instance of the development of a comprehensive machine learning model by integrating pretreatment brain functional measures from simultaneous PET/MRS in one framework.
- The pretreatment simultaneous PET/MRS used in the study takes around 60 minutes and has the potential to prevent weeks of failed treatment trials.
- Applying predictive measures from neuroimaging can reduce delay to effective treatment, patient suffering and economic burden, and enhance long-term functional outcomes.
- The findings may assist clinicians with treatment selection and shed light on the neurobiology of remission.

## 2. Material and methods

### 2.1. Study cohort

This study involved the analysis of simultaneously acquired PET/MRS neuroimaging data from a randomized, placebo-controlled, double-blind, single-site SSRI (Escitalopram) trial on 60 participants with MDD before initiating treatment. The study design and rationale for the data set have been previously described [6,10]. 43 regional measures of metabolic rate of glucose (MRGlu, mg/(min*100 mL)) from FDG-PET across multiple brain regions [6], and concentrations (mM) of Glx (Glu+Gln, a composite peak formed by Glu and Gln), GABA and the ratio of Glx to GABA (Glx/GABA)) in the ACC from MRS [10] were quantified as described in prior publications. After eight weeks of treatment, those with ≤ 7 on the 17-item Hamilton Depression Rating Scale were designated *a priori* as remitters (free of depression).

### 2.2. Machine learning analysis

**Fig. 1.**
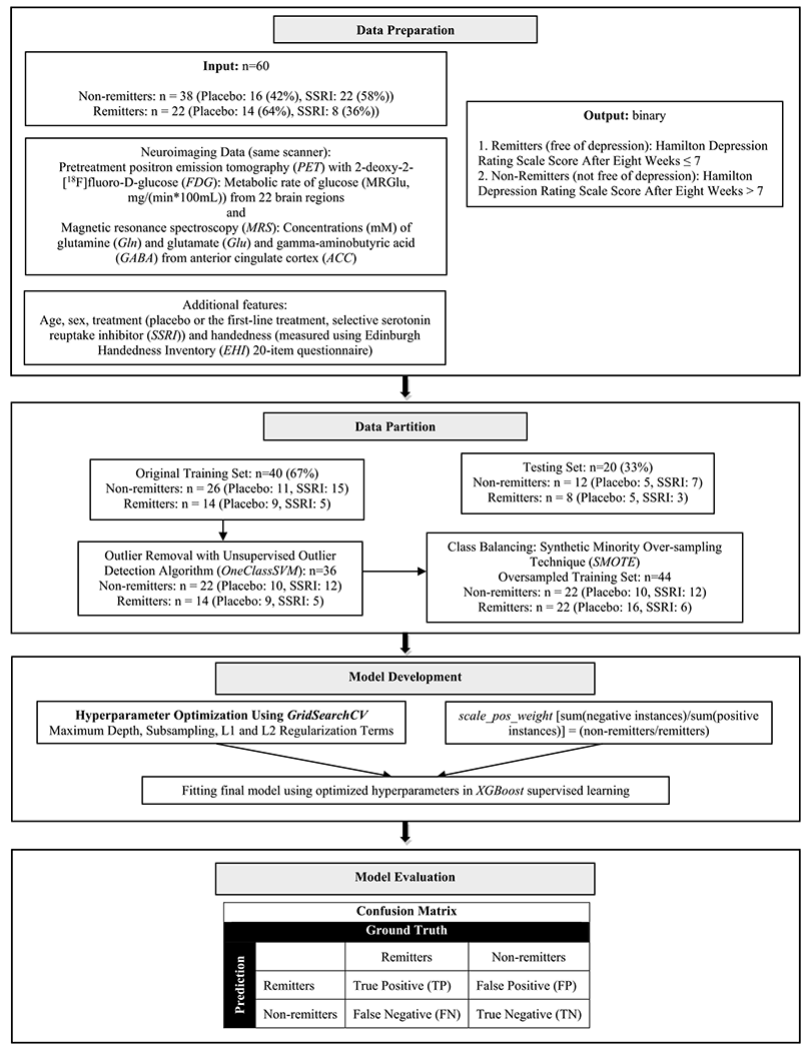
XGBoost model development and evaluation.

The ML model development with hyperparameters is illustrated in Fig. 1. The data partition was performed by random splitting into 67% train and cross-validation (*n* = 40), and 33% test (*n* = 20) sets, stratified by outcome (remitters vs. non-remitters) and treatment assignment (SSRI vs. placebo), to ensure comparable distribution in each data set. There is no established power calculation for XGBoost, so the conventional ML practice of evaluating the fitted model on validation set was used. The hyperparameter, *scale_pos_weight* [sum(negative instances)/sum(positive instances)] = (non-remitters/remitters) from the train set assigned greater weight to prediction of remitters. *OneClassSVM* identified and removed outliers from the train set. *Synthetic Minority Oversampling Technique* (*SMOTE*) oversampled the remitters class [15].

**Fig. 2.**
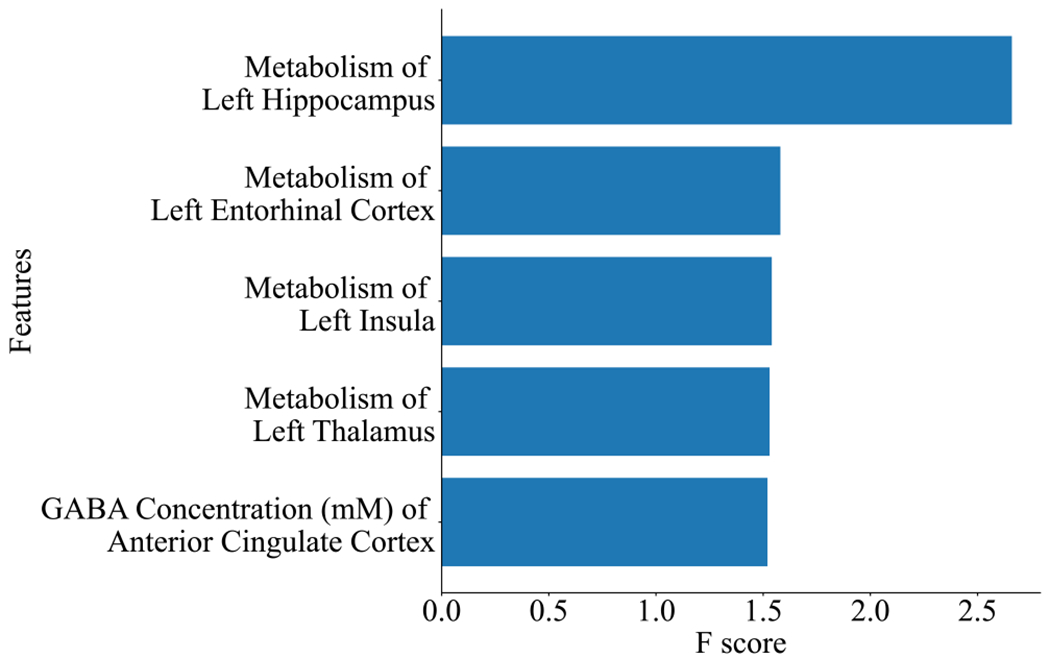
The most predictive imaging features from the XGBoost model. The features are as follows: metabolism estimated by the metabolic rate of glucose (MRGlu, mg/(min*100 mL)) of left hippocampus, left entorhinal cortex, left insula, left thalamus and GABA (*γ*-aminobutyric acid) concentration of anterior cingulate cortex. F score: relative contribution of the feature to the prediction model.

The hyperparameters for subsampling, number of trees, and depth of tree were optimized using *GridSearchCV* with 3 repetitions of stratified 10-fold cross-validation (preferred for depression research [16,17]). The 50 input features including the 46 imaging (43 PET and 3 MRS) measures, and information on age, sex, handedness, and treatment assignment were entered into XGBoost for training with optimized hyperparameters to predict remitters vs. non-remitters. The model performance was evaluated on the test data using confusion matrix (Fig. 2). Statistical and machine learning analyses were performed using STATA/SE 13.0 (StataCorp LLC, College Station, TX) and Python 3.9.0 (Python Software Foundation, Beaverton, OR).

## 3. Results

### 3.1. Study cohort

The study consisted of 60 participants with an age range of 18 to 64 years (mean ± standard deviation: 30 ± 14 years). 37 (62%) were females, 30 (50%) were placed on SSRI and 51 (85%) were right-handed. After eight weeks of treatment, 22 participants remitted (37% remitters) and 38 participants did not (63% non-remitters). There was no significant difference between the non-remitters and remitters groups in the study sample in terms of age, sex, handedness, or treatment assignment.

### 3.2. Machine learning analysis

**Fig. 3.**
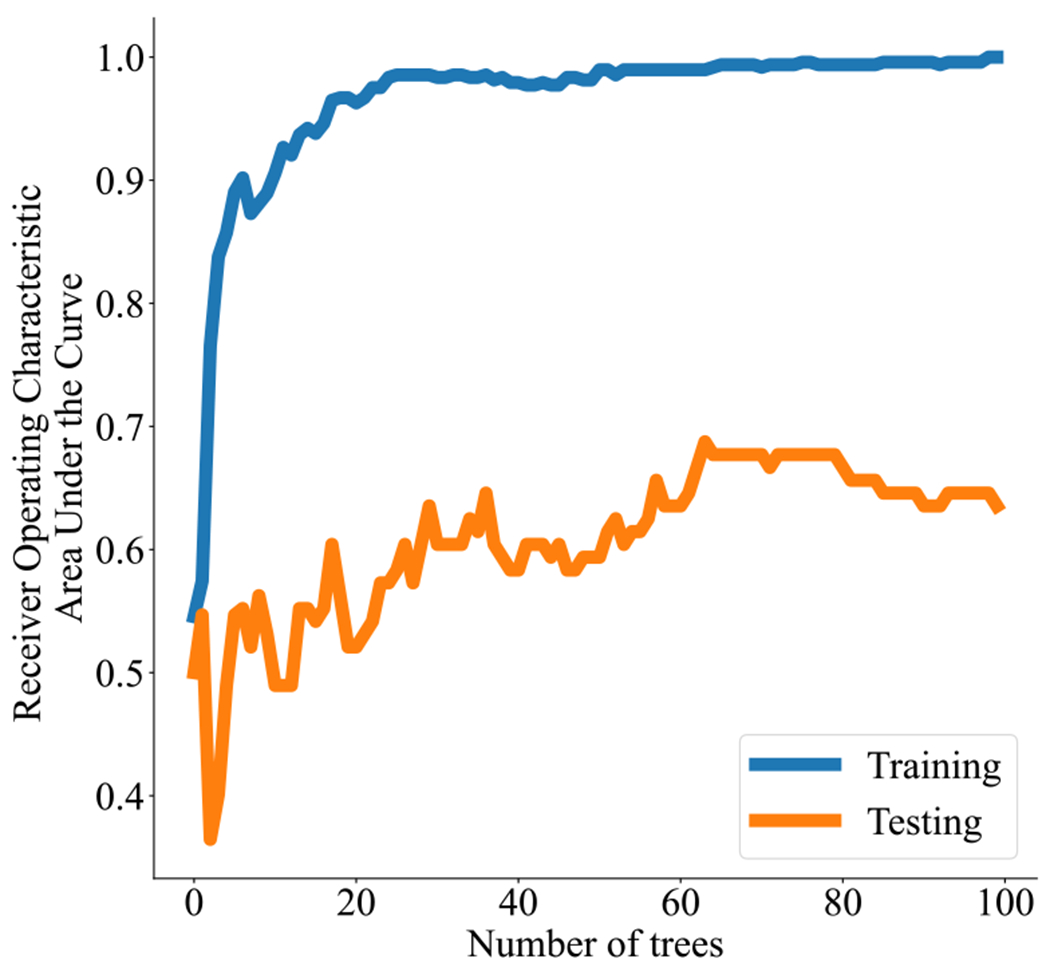
XGBoost receiver operating characteristic area under the curve.

In the training set (*n* = 40), there were 14 remitters (35%) with 5 on SSRI and 9 on placebo, and 26 (65%) non-remitters with 15 on SSRI and 11 on placebo (*scale_pos_weight* = 1.86). The most predictive neuroimaging features based on “gain” (according to their contribution to the fitted model) are shown in Fig. 2. The cross-validated training and testing Receiver Operating Characteristic (ROC) Area under the Curves are shown in Fig. 3.

Supplemental Table 1 and Table 1 below show the performance of the fitted model on the unseen test data (*n* = 20) consisting of 8 remitters (40%) with 3 on SSRI and 5 on placebo, and 12 (60%) non-remitters with 7 on SSRI and 5 on placebo.

## 4. Discussion

### 4.1. Novelty

This study was the first effort to build a comprehensive predictive model using simultaneous PET/MRS data from randomized clinical trial for prediction of remission. This was also the first time a gradient boosting decision-tree-based algorithm was used for this purpose. In this novel architecture, the XGBoost hyperparameters were utilized following oversampling and outlier removal, which resulted in the current model’s weighted accuracy (77%) comparable to previous studies on the prediction of antidepressant treatment outcome with much larger sample size [17–20].

### 4.2. Generalizability

The splitting in the current study ensured an adequate test set (as opposed to the alternative practice of 80/20 or 90/10 train/test split) to protect against performance misestimation in MDD research [21]. This model’s generalizability is further strengthened through *Stratified Cross Validation* which is particularly useful for analyzing small data sets with unbalanced classes, as seen in our data set with the remitters and non-remitters class. This technique allows similar proportion of different classes in each fold to ensure all strata of the data is well represented.

### 4.3. Regularization

Instead of feature reduction, the current model optimized regularization hyperparameters that ensures higher accuracy and better uncertainty assessment [22]. These XGBoost regularization hyperparameters, a.k.a. penalty terms *alpha* (L1, *LASSO Regression*) and *lambda* (L2, *Ridge Regression*), shrink the coefficients of less relevant features toward 0 [23]. Using this technique, this model achieved accuracy higher than a previous model with 59% accuracy that used feature reduction to select 25 most predictive variables for remission after 12 weeks of SSRI treatment from 164 patient-reportable variables [18]. Including more features also protects the predictive model performance from being affected by influential data points [19].

### 4.4. Performance

The true positive rate and true negative rate for classification in the held-out test data are reasonably high compared to the negligible values of false positive rate and false negative rate, attesting the acceptable performance of the model. At least 100 number of trees were used following convention when searching for the optimal number of trees, however the Receiver Operating Characteristic Area Under the Curve indicates that having a lower number of trees (~ 70) might have given slightly better performance.

### 4.5. Limitations

To address limitations related to small sample size in neuroimaging research, two most common data augmentation techniques for image classification including generative adversarial networks and unity game engine can be explored in future. However, this will require finding the optimal data augmentation strategy and developing evaluation systems to ensure quality of augmented data sets, while accounting for the computational cost for slower convergence.

### 4.6. Potential biomarkers

Even though there is a scarcity of predictive analytics using biomarkers with machine learning in neuropsychiatry, the insights from successful machine learning applications in neurological disorders such as stroke may be useful, considering the bidirectionality of stroke and depression [24]. The current model has been compared to predictive analytics using biomarkers from other modalities in Table 2. One such potential biomarker could be electromyography (EMG), since motor activity can be lower in depression [25–29], and increase with depression improvement [25,30,31]. The tree-based algorithm, random forest with real-time signals from thighs and calves of 287 participants has shown predictive accuracy > 90% for stroke [32]. Furthermore, electroencephalography (EEG) has been used for predicting stroke with Classification and Regression Trees (C&RT) algorithms with 89% accuracy [33]. With tree-based models such as C5.0 and random forest, EEG data has shown only around 70% accuracy for predicting stroke [34]. Pretreatment EEG measures have been useful for predicting antidepressant efficacy with > 87% accuracy using a mixture of factor analysis (MFA) classifier [1]. However, the biosignals received from EEG electrodes placed on the surface of the brain are less precise for locating the source of the signal as compared to PET/MRS used in the study [2]. Nonetheless, adding these cost-effective modalities to molecular neuroimaging may help develop a more comprehensive predictive model with improved sensitivity for predicting antidepressant response.

## 5. Conclusions

To our knowledge, this was the first effort to develop a gradient tree boosting classifier by integrating pretreatment multimodal molecular neuroimaging with easily interpretable brain functional measures in one framework, with accuracy comparable to previous predictive models. This study provides information on effectively addressing common issues related to neuroimaging analysis, such as small sample size, high dimensionality, and class imbalance. More importantly, the pretreatment neuroimaging takes around 60 minutes and has the potential to prevent weeks of failed treatment trials.

## Supplementary Material

Supplementary Material

## Figures and Tables

**Table 1:** The performance metrics of the fitted model on test data.

Performance Metrics	Recall/Sensitivity/True Positive Rate	False Negative Rate	Specificity/True Negative Rate	False Positive Rate	Weighted Accuracy	Precision/Positive Predictive Value	Negative Predictive Value	f1-Score	Sample Size
Overall	62%	38%	92%	8%	77%	83%	79%	0.71	20
Stratified by Treatment Assignment (SSRI vs. Placebo)									
SSRI	67%	33%	86%	14%	76%	67%	86%	0.67	10
Placebo	60%	40%	100%	0%	80%	100%	71%	0.75	10
Stratified by Sex (Female vs. Male)									
Female	50%	50%	100%	0%	75%	100%	75%	0.67	10
Male	75%	25%	83%	17%	79%	75%	83%	0.75	10

**Table 2:** A comparative table on the advantages and disadvantages of the proposed model.

Study	Advantages	Disadvantages
Hussain et al., 2020 [35]	Cost-effective for using EMG to address motor activity changes with antidepressant response	Accuracy lower than the current study using neural network model that requires relatively large sample
Yu et al., 2022 [32]	90% prediction accuracy with random forest	Lack of insight from neurobiology if only using EMG signals, but useful for multimodal analysis
Hussain et al., 2020 [33]	Portable EEG device for data collection, comparison of different algorithms	Removal of features before training the algorithm may lead to loss of information
Hussain et al., 2021 [34]	Wireless EEG devices used for data collection	Expertise will be required for EEG data interpretation and tree-based algorithms showed 70% accuracy

## Data Availability

The data used in the study can be provided if required on request from the corresponding author.
