# Supplementary material for: Gradient boosting decision-tree-based algorithm with neuroimaging for personalized treatment in depression
Source: Neurosci Inform. Author manuscript; Available in PMC 2023 Jan 24. (PMC9873411; doi:10.1016/j.neuri.2022.100110)
Supplement: Supplementary Material [file NIHMS1864649-supplement-Supplementary_Material.docx]

| Ground Truth | | | |
| --- | --- | --- | --- |
| Prediction |  | Remitters | Non-remitters |
|  | Remitters | TP=5 | FP= 1 |
|  | Non-remitters | FN=3 | TN=11 |

**Supplemental Table 1. Confusion Matrix for XGBoost Model Evaluation on Test Data.** From left to right: the confusion matrix shows true positive (TP, top left), false positive (FP, top right), false negative (FN, bottom left), and true negative (TN, bottom right) values used to calculate the performance metrics.

*Calculations:*

The true positive (TP), false positive (FP), false negative (FN), and true negative (TN) values from the above Supplemental Table 1 was used to calculate the following performance metrics.

$$Recall or Sensitivity or True Positive Rate =\frac{TP}{TP + FN}$$

$$False Negative Rate =\frac{FN}{TP+FN}$$

$$Specificity or True Negative Rate =\frac{TN}{TN + FP}$$

$$False Positive Rate=\frac{FP}{TN+FP}$$

$$Weighted Accuracy = \left( \frac{\left( \frac{TP}{\left( TP+FN \right)} \right)+\left( \frac{TN}{\left( TN+FP \right)} \right)}{2} \right)*100\%$$

$$Precision or Positive Predictive Value =\frac{TP}{TP + FP}$$

$$Negative Predictive Value =\frac{TN}{TN + FN}$$

$$f1-Score =2*\frac{\left( precision * recall \right)}{\left( precision + recall \right)}$$

$f1-Score$ calculates the harmonic mean, instead of the simple average. If the dataset $x_{1}, \ldots, x_{n}$ has associated weights $w_{1}, \ldots, w_{n}$ then its weighted Harmonic Mean, H = $\sum_{i=1}^{n} w_{i}/\sum_{i=1}^{n} \left( \frac{w_{i}}{x_{i}} \right)=\left( \frac{\sum_{i=1}^{n} w_{i}x_{i}^{-1}}{\sum_{i=1}^{n} w_{i}} \right)^{-1}$

The harmonic mean penalizes extreme values of precision and recall. The overall f1 score decreases as precision or recall reaches extreme values. For example, if our precision is 0.1 and recall is 0.8, the f1 score would be 0.18, which is a low value due to the low precision value. However, the simple average would be 0.45.
